# Advances in Personalized Nursing Care

**DOI:** 10.3390/jpm13121635

**Published:** 2023-11-23

**Authors:** Manuel Lopes, Luís Sousa, César Fonseca

**Affiliations:** 1 Nursing Department, University of Évora, 7000-801 Évora, Portugal; mjl@uevora.pt (M.L.); cfonseca@uevora.pt (C.F.); 2 Comprehensive Health Research Centre (CHRC), University of Évora, 7000-801 Évora, Portugal; 3 Nursing Department, Escola Superior de Saúde Atlântica, 2730-036 Barcarena, Portugal

Patient-centered care reflects the quality of personal, professional, and organizational relationships. In this sense, efforts are made to promote care centered on the patient and their family [1]. The provision of health services must meet the needs of patients and caregivers as it is essential to promote positive care outcomes and perceptions of care quality, complying with the principles of patient-centered care [2]. As a result, patients are more active when interacting with healthcare professionals during care. Universities have an important role in training health professionals to be attentive, informative, and empathetic, with the objectives of promoting partnership, solidarity, empathy, and patient participation [1].

The active participation of patients in their care processes has been recognized internationally to improve the safety and effectiveness of care. Individualization in such processes requires engagement, involvement, or participation, characterized by interactive actions both on the part of the patient, such as asking questions, speaking, acquiring knowledge, learning, and decision making, and on the part of the nurse, namely in terms of recognizing, responding, sharing information, teaching, and collaborating [3].

From this perspective, the person-centered care model ensures that the person’s rights, regardless of their age and functionality, are respected. This model considers the person as the center of the care process, in which personalized care is provided, having positive impacts for both patients and healthcare professionals.

In this Special Issue, we present twenty articles [4,5,6,7,8,9,10,11,12,13,14,15,16,17,18,19,20,21,22,23], eleven primary articles [4,5,6,7,8,9,10,11,12,13,14], six literature reviews [15,16,17,18,19,20], and three protocols [21,22,23] providing a comprehensive overview of advances in personalized nursing care throughout the life cycle and in various care environments, such as intensive care, chronic disease care, oncology care, rehabilitation, and community care.

The included studies explore situations of greater vulnerability as well as care for health professionals [15,16].

Researchers from around the globe participated in this Special Issue, representing countries such as Portugal, Spain, Saudi Arabia, Brazil, Canada, Italy, Poland, Finland, Sweden, Croatia, and the Netherlands.

The research presented herein emphasizes and reinforces the contribution of patient-centered care to the health and well-being of people experiencing illness and vulnerability, as well as their families.

We consider the findings from this collection to have implications for improving personalized nursing care, nursing education, and nursing management and the construction of education and health policies.
